# Physico-thermal and emission properties of tissue cultured clone from *Bambusa balcoaa* (Beema bamboo) and *Oxytenanthera abyssinica* as sustainable solid biofuels

**DOI:** 10.1371/journal.pone.0279586

**Published:** 2022-12-22

**Authors:** Kwadwo Boakye Boadu, Michael Ansong, Rogerson Anokye, Kelvin Offeh-Gyimah, Enoch Amoah

**Affiliations:** 1 Department of Wood Science and Technology, Faculty of Renewable Natural Resources, Kwame Nkrumah University of Science and Technology, Kumasi, Ghana; 2 Department of Silviculture and Forest Management, Faculty of Renewable Natural Resources, Kwame Nkrumah University of Science and Technology, Kumasi, Ghana; Universiti Teknologi Petronas: Universiti Teknologi PETRONAS, MALAYSIA

## Abstract

In the search for alternatives to wood fuel, to meet the bio-energy requirement of an ever-increasing global population, the International Network for Bamboo and Rattan has supported farmers in many tropical countries to establish plantations of Beema bamboo (a tissue-cultured clone from *Bambusa vulgaris*) and *Oxytenanthera abyssinica* for bio-energy production. The quality of these species as solid biofuels is unknown due to the absence of data on their physico-thermal and emission characteristics. Using the American Standard for Testing and Materials and other internationally accepted standards, their ultimate and proximate analysis, and physico-thermal and emission properties were evaluated. Beema bamboo and *O*. *abyssinica* have high Hydrogen, organic and fixed Carbon contents and low quantities of ash, moisture content, volatile matter, Oxygen, Nitrogen and Sulphur. This will contribute to their heating values and low oxide emissions. Based on their High Heating Values (Beema bamboo = 23.22 MJ/kg; *O*. *abyssinica* = 23.26 MJ/kg), the species will be suitable for high energy-using applications. The Particulate Matter and Carbon Monoxide concentrations (Beema bamboo: 90 ug/m^3^ and 2.83 ppm respectively; *O*. *abyssinica*: 77.33 ug/m^3^ and 3.20 ppm respectively) are lower than the threshold (35000 ug/m^3^ and 9 ppm respectively) approved by the United States Environmental Protection Agency. These properties make the species good raw materials for solid biofuel which is safe for indoor use. Their use will contribute to reducing pressure on tropical forests for wood fuel and the health hazards associated with fossil fuel use.

## Introduction

Limited access to energy supply is a barrier to sustainable development [1]. In 2019, the International Energy Agency [2] reported that some 2.6 billion people did not have access to clean energy for cooking. This represented about one-third of the global population. Currently, it is estimated that about three billion people remain without clean fuel for domestic purposes. As the global population continues to rise, so will the demand for energy. According to [3], global energy consumption is expected to rise by 56% between 2010 and 2040. The global economy’s over-reliance on fossil fuels in the past has led to an increase in greenhouse gas emissions with consequences on the world’s climate system. It is in this regard that the United Nations Sustainable Development Goal 7 seeks to improve global access to inexpensive and consistent energy provision. It hopes to substantially increase the renewable energy share of the global energy mix. This would contribute to climate change mitigation.

Bioenergy is the single largest renewable energy source which supplies about 10% of the world’s primary energy needs [4]. Generally, biomass fuels provide about 35% of energy supplies in developing countries [5]. In Africa, about half of the domestic energy demand is met using bioenergy in the form of fuelwood [6]. About 50 million tons of wood are harvested from tropical forests every year to meet the energy requirement of some developing countries in sub-Saharan Africa. With rapid industrialization and global population growth, fuelwood production is expected to increase. The production of wood fuel most frequently results in forest depletion [7]. Alternative sources of bioenergy supply besides fuelwood including crop residues, animal dung, and non-timber industrial materials like bamboo have been recommended [5]. Substitution of fuelwood with these alternatives will reduce pressure on natural forests.

The International Network for Bamboo and Rattan (INBAR) has made progressive efforts to promote the production of bio-energy from bamboo resources as a replacement for fuelwood [8]. Bamboo is one of the fastest-growing plants in the world. It is relatively easy to grow and maintain, has high renewability, and is a cost-effective substitute for energy production [9]. INBAR has engaged smallholder farmers in tropical countries in bamboo plantations (over 600,000 hectares) for biomass fuel production to bridge the global energy demand and supply gap. The species planted include Beema bamboo (a tissue-cultured clone from *Bambusa vulgaris*) and *Oxytenanthera abyssinica*, which originated from India and Ethiopia respectively. Boadu et al. [10] found that these species contain considerably high quantities of biomass (over 100,000 tons of biomass from 2000-acre plantation) which could make them excellent materials for bioenergy generation. However, the authors did not investigate the fuel characteristics and emission properties of the bamboo. The selection of biomass as fuel materials must be based on their physical, thermal and emission characteristics [11]. These are important for the efficient and safe utilization of biomass. Unfortunately, this information is not available for Beema bamboo and *O*. *abyssinica*. Although data on the fuel characteristics of some other bamboo species are available in the literature, different bamboo species have different compositions, and this could influence their thermal and emission properties. Therefore, the specific physico-thermal and emission properties of these new bamboo species need to be ascertained. This is important to promote the development of Beema bamboo and *O*. *abyssinica* as sustainable solid fuel materials. Also, previous works on the fuel characteristics of bamboo failed to characterize the emission properties of the species they worked on which are needed to safeguard the health of the users of the fuel.

The moisture content and density, and burning rate and calorific value (High heating value and Low heating value) are respectively the physical and thermal properties of biomass, which are important for assessing the quality of solid biofuels ([11, 12]). They affect the rate of heating and drying of biomass during combustion. These, together with the ultimate (i.e., nitrogen, hydrogen, oxygen and organic carbon content) and proximate (ash, volatile matter and fixed carbon contents) properties influence the total amount of energy that is available in the fuel [13]. The combustion of biomass sometimes releases toxic gases such as particulate matter (PM_2.5_ in μg/m^3^) and carbon monoxide (CO in ppm), which are harmful to the environment and human health [14]. Millions of deaths occur each year from emissions released during biofuel combustion [1]. Therefore, many authors have recommended that the emission properties of biomass must be critically examined before their selection since they give an indication of how safe the biofuel material is to the environment and human health.

In the current study, the bioenergy potential of Beema bamboo and *O*. *abyssinica* was evaluated. Specifically, the physical, thermal, ultimate, proximate and emission properties of the species were determined. This information would be necessary for the promotion of the species as sustainable solid biofuel materials in the renewable energy production matrix.

## Materials and methods

### Harvesting and processing of bamboo

Six bamboo culms each of Beema bamboo and *Oxytenanthera abyssinica* were randomly harvested from private plantations (about 200 hectares) established in Jeduako in the Ashanti region of Ghana (Coordinates: longitude −1.9388⁰ west and latitude 6.3152⁰ north). The authors were introduced to the farmers who are the owners of the plantation by officials of INBAR and the appropriate permission was sought from the farmers before the bamboo was harvested. The site lies in the moist semi-deciduous forest zone dominated by sandy loam soil. The zone has an average annual rainfall of 1,270 mm. The highest amount of rainfall is often recorded in May/June and September/October. The temperature typically varies from 20°C to 33°C.

The culms were cleaned of dust, debris and sheaths and processed into different sizes for the various tests. The samples were air-dried for a week.

### Determination of the physical properties of *O*. *abyssinica* and Beema bamboo

#### Moisture content

Clean and dry petri dishes (Manufacturer: ZHIBANG; Model: ZB-PD90) were weighed. Following the ASTM E1756-08 standard, about 10 g of ground samples from each of the bamboo species were put into separate petri dishes using a spatula. The dishes with the samples were quickly weighed, oven-dried at 103±2 ⁰C for 2 h, transferred into a desiccator (Manufacturer: As ONE/Others; Model: 120) to cool and the final weight determined. The test was repeated 3 times. The moisture content (MC) of the samples was then calculated:

$$MC(\%)=\frac{W1-W2}{W1}\times 100$$ (1)

*W₁* = Weight of sample before oven-drying; *W₂* = Weight of sample after oven drying.

#### Density

The ASTM D2395B standard was followed in the determination of the density of the bamboo samples. The initial mass of a 3 cm long air-dried culm from each of the species was measured. The culms were tightly wrapped in polyethylene bags and submerged in a beaker with a known volume of water. The change in volume of water was recorded and the volume of the specimen was taken as the volume of water displaced. The density was then calculated from the mass and volume of the culms. The test was repeated 3 times.

### Ultimate analysis of *O*. *abyssinica* and Beema bamboo

#### Organic carbon

The organic carbon content of the species was determined using Walkely-black oxidation method. Potassium dichromate solution (0.1667 N) was first prepared by dissolving 49.04 g of reagent grade potassium dichromate dried at 105°C in 1000 ml of distilled water. Ferrous sulfate solution (0.5N) was also prepared by dissolving 278.02 g of ferrous sulphate in 500 ml distilled water and adding 40 ml of conc. H_2_SO_4_. Diphenylamine indicator was prepared by dissolving 0.5 g of diphenylamine in a mixture of 100 ml conc. H_2_SO_4_ and 20 ml distilled water. About 0.1 g of ground sample of each species was weighed into an Erlenmeyer flask (Manufacturer: Corning Incorporated; Model: 4980-2L) and 10 ml of 1.0 N K_2_Cr_2_O_7_ solution and 20 ml of conc. H_2_SO_4_ were sequentially added. The mixture was swirled and allowed to stand for 30 min for complete digestion. About 200 ml of distilled water, followed by 10 ml orthophosphoric acid and 2 ml diphenylamine indicator were added after digestion. The solution was titrated against 0.5 N ferrous sulphate solution until the colour changed to dark blue and then to a green end point. The titre values for the blank and sample solutions were respectively recorded. The test was repeated 3 times. The organic carbon content (C) of the samples was then calculated:

$$C(\%)=\frac{N\times (Vbl-Vs)\times o.oo3\times 1.33\times 100}{g}$$ (2)

*N* = Normality of ferrous sulfate = 0.5 N; *Vbl* = titre value of blank solution; *Vs* = titre value of sample solution; *g* = mass of sample taken; 0.003 = milli-equivalent weight of C in grams (12/4000); 1.33 = correction factor.

#### Hydrogen content

The titrimetric method was used to evaluate the hydrogen content of the samples. About 3 g of the air-dried ground sample were put into a digestion flask. Aqua-regia (10 mls) was added and digested for 10 mins. The content of the flask was filtered into 100 ml volumetric flask and diluted with distilled water. About 10 mls of the digest in the volumetric flask was measured into the Erlenmeyer flask and 5 drops of phenolphthalein indicator was added. The digest was titrated with 0.05 N NaOH to pink end point and the volume (ml) of NaOH used (V) was recorded. The test was repeated 3 times. The hydrogen content of the sample was calculated:

$$H(\%)=\frac{V*0.05*100}{W}=V*1.67$$ (3)

*V* = Titre volume of NaOH used (ml); Normality of NaOH = 0.05 *N*; *W* = weight of air-dried sample used

#### Oxygen content

The oxygen content of the biofuel was calculated based on the formula by Viotto et al. [15] as follows:

O(%)=100−C−H−Ash−N−S (4)

*O* = Oxygen content; *C* = Carbon; *H* = Hydrogen; N = Nitrogen; S = Sulphur

### Nitrogen content

The nitrogen content was determined using Kjeldahl method. About 1 g of the ground samples was oven-dried and cooled in a desiccator. It was put in a 500 ml long–necked Kjeldahl flask (Corning 5420–100) and 10 ml of distilled water was added. The mixture was left for 10 min to moisten. One spatula full of Kjeldahl catalyst [mixture of l part Selenium + 10 parts CuSO_4_ + 100 parts Na_2_SO_4_] [16] was added to the mixture after which 10 ml conc. H_2_SO_4_ was also added. The mixture was digested until it was clear and colourless or light greenish (1–1.5 h). The flask was cooled and the decant digested into a 50 ml volumetric flask with distilled water used to rinse the digestion flask. An aliquot of 10 ml of the digest was transferred by pipetting into the Kjeldahl distillation apparatus and adding 90 ml of distilled water. About 20 ml of NaOH was then added. The distillate was collected over 10 ml of 4% Boric acid and 3 drops of mixed indicator in a 250 ml conical flask. About 100 ml of the distillate was titrated with 0.l N HCl till the blue color changed to grey and suddenly to pink. The test was repeated 3 times. The nitrogen content (N) was calculated as:

$$N(\%)=\frac{(a-b)\times 1.4\times N\times V}{s\times t}x\;100$$ (5)

*a* = HCl used in the sample titration; *b* = HCl used in the blank titration; *N* = Normality of standard HCl; *V* = total volume of digest; *S* = mass of oven dried sample taken for digestion; *t* = volume of aliquot taken for distillation (10 ml)

#### Sulphur content

The Sulphur content of the bamboo samples was determined using the Spectrophotometer method. Serial standards of 5, 10, 20, 30, 40 and 50 mg/L were prepared from a pure sodium sulphate compound. About 5% of Barium chloride (BaCl) from a pure compound and 0.5% of gum acacia-acetic acid (GAAA) were individually prepared and 2 ml of each serial standard was pipetted into labelled test tubes. About 2.0 ml of the sample mixture were also pipetted into labelled test tubes. In each tube, 0.5 ml of GAAA and 1.0 ml of BaCl were sequentially added to the sample in each tube. The tubes were incubated at room temperature for 30 mins. The turbidity intensity on the spectrophotometer at 420 nm was recorded. A calibration curve from the serial standards and their corresponding optical densities was plotted and the level (concentration) of sulfur in the samples determined. The test was repeated 3 times.

### Proximate analysis of *O*. *abyssinica* and Beema bamboo

#### Ash content

The ash content of the samples was determined using the test protocol ASTM D1102-84 (2021). A crucible was oven-dried at 150 ⁰C and 5 g of ground samples was put into it and weighed. The sample was carefully placed in the muffle furnace and heated at 700 ⁰C for 2 h. The crucible with the ash was placed in a desiccator to cool and then weighed. The test was repeated 3 times. Ash content was calculated as follows:

$$Ash\;content(\%)=\frac{w2-w1}{w3-w1}\times 100$$ (6)

*w*1 = weight of empty crucible; *w*2 = weight of crucible with ash; *w*3 = weight of crucible with ground sample.

#### Volatile matter

The volatile matter content of the bamboo was determined based on European Standard EN15148-2009. Ground air-dried samples (1 g) were put in a crucible of known weight and covered. The crucible was heated in a furnace at 900°C in the absence of air for 7 mins. The crucible was taken out, cooled in a desiccator, and immediately weighed to determine the weight loss due to devolatilization. The loss in weight of the sample in the crucible represented the volatile matter content of the sample. The test was repeated 3 times.

#### Fixed carbon content

The fixed carbon content was determined from the difference between 100 and the sum of the percentages of volatile matter, ash, and moisture [17].

### Determination of the thermal properties of *O*. *abyssinica* and Beema bamboo

#### Calorific value

The methods described by Antwi-Boasiako and Glalah [11] was used to determine the calorific value of ground bamboo samples using Oxygen Bomb Calorimeter (model: XRY-1A; measurement accuracy: +/- 60j/G). The measurement was done at a constant pressure of 3 MPa. The initial temperature of the distilled water in the calorimeter was 25°C. The temperature of the exhaust gas was maintained above 300°C during the test. A computer attached to the calorimeter recorded and displayed the calorific values (Higher Heating Values /HHV) of the samples during the test. The test was repeated 3 times. The Lower Heating Values (LHV) were calculated from the formulae:

LHV=HHV−10.55(Moisturecontentofmaterial−9xHydrogencontentofmaterial) (7)

#### Burning rate

The bamboo culms (6 cm long) were weighed, supported horizontally at one end by a clamp and the free end ignited by a Bunsen burner for 30 sec. The burner was removed and the time taken by each sample to burn to ash was noted. The burning rate was obtained from the ratio of the mass of the sample in grams and the time taken by the sample to burn to ash in minutes [11]. The test was repeated 3 times.

### Determination of emission properties of *O*. *abyssinica* and Beema bamboo

The concentrations of Carbon Monoxide (CO) and Particulate Matter (PM_2.5_) emitted from Beema bamboo and *O*. *abyssinica* during combustion were determined using the gasification method. Pre-weighed air-dried bamboo culms were ignited and inserted into a wok stove (length = 80 cm, width = 70 cm, height = 65 cm) with an iron pot in the middle. The smoke from the combustion entered into a mixing chamber (volume = 0.20*m*^3^, distance from the grate to the bottom of the iron pot = 30 cm, distance from the grate to the ground = 15 cm) in which a built-in fan continuously mixed the smoke that exited and minimized the influence of temperature on the samples [18]. The concentrations of CO and Particulate Matter were measured during the combustion using CO data logger (at 10 sec interval) and UCB PM meter (once every minute) respectively for 1 hr. Both instruments were fixed 1.5 m above and 1 m to the side of the stove. The test was repeated 3 times.

### Data analysis and presentation

The data obtained from the tests were subjected to ANOVA at 5% significance level to determine the differences that exist in the physico-thermal and emission properties of the two species. The data were presented in charts and tables.

## Results and discussion

### Physical properties of *O*. *abyssinica* and Beema bamboo

#### Moisture content

The difference in the moisture content of the two materials was not significant (p >0.05). Biomass with high moisture burn less readily; much of the energy is used to heat and vapourize the water resulting in less heat per unit mass. Therefore, low moisture levels in biomass are often preferable ([19, 20]). The optimum moisture content that ensures quality solid biofuels is approximately 8 to 10% [21]. In a study by Purbasari et al. [20] on the thermal characterization of *Gigantochloa apus*, *Gigantochloa levis* and *Gigantochloa atroviolacea* as solid biofuel raw materials, the recorded moisture contents ranged between 8.13% and 8.89%. Based on moisture content, these species were recommended as good biomass resources for fuel since they will be easily combustible. Similarly, the low moisture content recorded for Beema bamboo (7.6%) and *O*. *abyssinica* (7.3%) after air-drying for a week will promote their efficient combustion as solid biofuels.

#### Density

The densities of Beema bamboo and *O*. *abyssinica* differed significantly (p ≤ 0.05). The density of bamboo generally ranges between 0.4 g/cm^3^ and 0.9 g/cm^3^ [22]. Our values are within this range. The density of biomass affects the level of energy production, rate of heating and drying during combustion ([23, 24]). Rusch et al. [25] found *Bambusa vulgaris*, *Phyllostachys aurea and Dendrocalamus asper* to have good heating properties partly due to their densities (i.e., 0.602 g/cm^3^, 0.647 g/cm^3^, and 0.543 g/cm^3^ respectively). Shojaeiarani et al. [3] also reported an average heating value of 30.2 MJ/kg (30,200 kJ/kg) for several unnamed wood and attributed this value to their densities (0.42–0.67 g/cm^3^). Based on density, Beema bamboo and *O*. *abyssinica* will equally have good heating values. Their less dense nature will reduce their handling and storage costs.

### Ultimate analysis of *O*. *abyssinica* and Beema bamboo

#### Organic carbon

Organic carbon in *O*. *abyssinica* than Beema bamboo was not significantly different (p > 0.05). Shojaeiarani et al. [3] concluded that the carbon content of various biomass used as solid biofuels ranges between 42.2% and 51.8%. Generally, the organic carbon content of bamboo is over 45% ([26–28]). Park et al. [29] found the carbon content of varieties of Indonesian bamboo recommended as fuel resources to be 48.2%– 50.9%. This is lower than the values we recorded for Beema bamboo (52.74%) and *O*. *abyssinica* (52.97%) in the current study. The relationship between carbon content of biomass and calorific value is well known; high carbon content results in high calorific value [30]. The high amount of carbon recorded in this study imply that both species will have high calorific values.

#### Hydrogen and oxygen content

While the hydrogen content was significantly different between *O*. *abyssinica* and Beema bamboo (p <0.05), the oxygen content was not significantly different (p >0.05). Heat is released when hydrogen is burnt to gaseous water. Therefore, high hydrogen percent in biofuels is desirable. On the contrary, low amount of oxygen is required because oxygen binds with carbon and hydrogen and consequently lowers the heating value of the biomass [31]. The percentages of hydrogen and oxygen in several solid fuel biomass ranges from 5.4 to 7.2% [3] and 37.75–75.72 ([31, 32]) respectively. Beema bamboo and *O*. *abyssinica* had comparatively high hydrogen content (9.95% and 10.45% respectively) and low oxygen content (33.27% and 32.98% respectively) (Table 1). This is favourable for the species for their use as solid biofuels.

**Table 1:** The physical and ultimate properties of *O*. *abyssinica* and Beema bamboo.

Properties	N	Minimum	Maximum	Mean	Std. Error	Std. Deviation
**Beema bamboo**						
Moisture content (%)	3	7.50	7.80	7.63	0.12	0.15
Density (g/cm^3^)	3	0.39	0.48	0.43	0.26	0.05
Organic carbon (%)	3	52.69	52.80	52.74	0.07	0.06
Hydrogen (%)	3	9.79	10.10	9.95	0.08	0.16
Oxygen (%)	3	32.90	33.97	33.27	0.28	0.60
Nitrogen (%)	3	0.68	0.71	0.69	0.01	0.02
Sulphur (%)	3	0.16	0.18	0.17	0.09	0.01
***O*. *abyssinica***						
Moisture content (%)	3	7.04	7.50	7.3	0.12	0.24
Density (g/cm^3^)	3	0.49	0.56	0.52	0.02	0.04
Organic carbon (%)	3	52.80	53.13	52.97	0.07	0.16
Hydrogen (%)	3	10.35	10.55	10.45	0.08	0.10
Oxygen (%)	3	32.32	32.88	32.98	0.28	0.54
Nitrogen (%)	3	0.58	0.59	0.59	0.01	0.01
Sulphur (%)	3	0.24	0.28	0.26	0.09	0.02

#### Nitrogen and sulphur content

Nitrogen proportion was significantly higher (p <0.05) in Beema bamboo than *O*. *abyssinica* (Table 1). The reverse was recorded for the Sulphur content (p <0.05). The complete oxidation of Nitrogen and Sulphur during fuel combustion is responsible for the emission of nitrogen and Sulphur oxides. These oxides result in increasing ozone, smog, and respiratory problems. Hence, low amounts of Nitrogen and Sulphur in biofuels are good indicators for biomass selection. Bamboo generally has a low Nitrogen and Sulphur content ([33–35]). The nitrogen and Sulphur content of Beema bamboo and *O*. *abyssinica* are 0.69% and 0.59% and 0.17% and 0.26% respectively. The combustion of biofuel from these species will lead to the emission of low Nitrogen and Sulphur oxides into the environment, reducing their impact on human health and the environment.

### Proximate analysis of *O*. *abyssinica* and Beema bamboo

#### Ash content

The ash content of biofuels mostly lies between 0.5% and 10% [36]. The values for Beema bamboo (3.17%) and *O*. *abyssinica* (3.05) (Table 2) are lower than the reported values. The ash content did not differ significantly between the two species (p >0.05). Ash is the inorganic non-combustible component of biomass that has the capability of lowering the calorific value and heat of combustion of fuel materials ([23, 24, 37]). Fuel with high ash content often produces high dust emissions and affects its combustion efficiency [38]. This is not desirable in solid biofuels. Birch wood and briquette were found to have ash contents of 18.3% and 10.7% respectively [39]. These ash content values were known to have great effect on the calorific value of the samples. Wheat straw and rape stalk were observed to have low ash content (9.37% and 6.42% respectively) by He et al. [40]. They recommended these materials for fuel production. The values recorded for Beema bamboo and *O*. *abyssinica* will contribute to their efficient combustion as solid biofuels.

**Table 2:** The proximate analysis, thermal and emission properties of *O*. *abyssinica* and Beema bamboo.

Properties	N	Minimum	Maximum	Mean	Std. Error	Std. Deviation
**Beema bamboo**						
Ash content (%)	3	2.40	3.65	3.17	0.34	0.67
Volatile matter (%)	3	59.98	61.61	60.84	0.34	0.82
Fixed carbon (%)	3	28.11	28.57	28.36	0.28	0.23
High Heating Value (MJ/kg)	3	23.05	23.48	23.22	0.11	0.23
Low Heating Value (MJ/kg)	3	22.04	22.45	22.19	0.11	0.22
Burning rate (g/min)	3	3.45	3.57	3.52	0.04	0.61
Particulate Matter (ug/m^3^)	3	86	95	90	2.78	4.58
CO (ppm)	3	2.5	3.1	2.8	0.23	0.31
***O*. *abyssinica***						
Ash content (%)	3	2.75	3.6	3.05	0.34	0.48
Volatile matter (%)	3	62.70	62.75	62.72	0.34	0.03
Fixed carbon (%)	3	26.20	27.46	26.93	0.28	0.65
High Heating Value (MJ/kg)	3	23.07	23.36	23.26	0.11	0.16
Low Heating Value (MJ/kg)	3	22.00	22.29	22.19	0.11	0.17
Burning rate (g/min)	3	2.40	2.56	2.49	0.04	0.08
Particulate Matter (ug/m^3^)	3	72	82	77.33	2.78	5.03
CO (ppm)	3	2.8	3.7	3.2	0.23	0.46

#### Volatile matter

There was a significant difference in the volatile matter content recorded for the two species (p <0.05). High volatile matter in biofuel is advantageous for gasification since they will volatilize and burn as gas during combustion [38]. However, for use as solid biofuels, low amount of volatile matter and high fixed carbon content are preferred in the raw material. High quantities of flammable substances (primarily CO, low hydrocarbons, and monocyclic aromatic hydrocarbons) are released during the combustion of high volatile matter fuels which are presented as high flames [41]. As such, supplementary air needs to be introduced to ensure effective combustion. The incomplete combustion of volatile matter produces dark smoke and results in heat loss, pollution and soot deposition on boiler surfaces [42]. Consequently, high volatile matter is unwanted for solid biofuels and great efforts are made to reduce its content in the biofuel before utilization. Smołka-Danielowska et al. [39] found that wood biomass is characterized by volatile matter content ranging between 69.3–81%. In a study on the production of solid biofuel from palm wastes, Yuliansyah et al. [43] found that palm fronds and trunks had very high volatile matter of 82.5% and 83.9% respectively, which were not good for solid biofuels. They subjected these materials to hydrothermal treatment at 200–350°C for 30 min in order to reduce the volatile matter content. This increases the cost of producing the solid biofuel. Fortunately, the volatile matter values recorded for Beema bamboo (60.84%) and *O*. *abyssinica* (62.72%) (Table 2) make them ideal raw materials for solid biofuel production.

### Fixed carbon content

There was a significant variation in the fixed carbon content of Beema bamboo and *O*. *abyssinica* (p <0.05). Fixed carbon content of biomass gives an indication of its heating value and acts as the main heat generator during combustion [23]. High fixed carbon in biomass is advantageous for the production of solid biofuels. Patel and Gami [42] investigated the fixed carbon content of coniferous tree bark, sawdust, wheat straw, miscanthus straw and poultry litter as biofuel materials. The fixed carbon content of these materials ranged from 7.2% to 48.7%. They indicated that the recorded fixed carbon content was favourable for the use of the materials as fuel. Based on fixed carbon content in Table 2, Beema bamboo and *O*. *abyssinica* will have good heating values.

### Thermal properties of *O*. *abyssinica* and Beema bamboo

#### Calorific value

There was no significant difference in the High Heating Value (HHV) and Low Heating Value (LHV) obtained for the two species (p >0.05 and p >0.05 respectively) (Table 2). Calorific value is a measure of the energy content of fuel. It is the basis for evaluating the quality of fuel as an energy source [41]. As already discussed, calorific or heating value strongly depends on the moisture content and elemental composition of the fuel. For instance, it increases with increasing Carbon and Hydrogen content, and decreasing Oxygen and ash content [40]. The HHV of Beema bamboo (23.22 MJ/kg) and *O*. *abyssinica* (23.26 MJ/kg) were higher than those obtained by Ediriweera [44] for different varieties of bamboo (18.27–20.54 MJ/kg or 18,270 kJ/kg– 20,540 kJ/kg). It was also higher than the values recorded for solid biofuels made from spent shiitake substrate and three types of biochar (6.5 to 18.1 MJ/kg or 6,500 kJ/kg to 18,100 kJ/kg; [15]) but lower than biofuel from palm frond and trunk (18–29.7 MJ/kg or 18,000–29,700 kJ/kg; [42]). The LHV was 22.19 MJ/kg for both Beema bamboo and *O*. *abyssinica* respectively. The high calorific value recorded in the present study could be attributed to the high carbon content and low ash content of the bamboo species. Based on their calorific values, the species will be suitable for high energy-demanding applications.

### Burning rate

Burning rate differed significantly between the species (p <0.05). Burning rate refers to the speed by which a given mass of fuel is consumed. High burning rate has the potential of creating an oxygen deficient condition that produces high pollutant emissions from incomplete combustion [18]. Therefore, biomass with high burning rates are less preferred as solid biofuels due to their quick combustion and thermal inefficiencies [11]. Shen et al. [18] classified burning rate of 0.029–0.064 kg/min (29–64 g/min) as slow to medium. The burning rates of Beema bamboo and *O*. *abyssinica* (3.52 g/min and 2.49 g/min respectively) (Table 2) could be classified as low. This makes the two species important resources for solid biofuel.

### Emission properties of *O*. *abyssinica* and Beema bamboo

There was no significant difference in the Carbon monoxide concentrations between the species (p >0.05). The difference for Particulate Matter was significant (p <0.05). The negative effects of biomass smoke exposure on human health and the environment have been extensively discussed ([7, 14, 45]). It is reported that about 1.5 million people die every year as a result of indoor air pollution [46]. Many more people suffer from lung cancer, pneumonia, stroke and ischaemic heart disease. These are mainly caused by high levels of particulate matter and carbon monoxide emissions while burning solid fuels [14]. Therefore, the choice of biomass fuel, especially for indoor use should be based on the fuel’s emission properties. According to United States Environmental Protection Agency [47], the permissible average Particulate Matter and Carbon monoxide concentrations for biomass fuels are 35 mg/m^3^ (35000 ug/m^3^) and 9 ppm respectively. The values recorded for Beema bamboo (90 ug/m^3^ and 2.83 ppm respectively) and *O*. *abyssinica* (77.33 ug/m^3^ and 3.20 ppm respectively) are lower than the allowable concentrations. Thus, these bamboo species are good fuel materials safe for indoor use. Beema bamboo will release more Particulate Matter than *O*. *abyssinica* during combustion.

## Conclusion

The current work evaluated the physico-thermal and emission properties of Beema bamboo and *Oxytenanthera abyssinica* as solid biofuels that could replace fuelwood in providing safe and efficient renewable bioenergy. Beema bamboo and *O*. *abyssinica* are less dense and have high Organic Carbon and Hydrogen content, and low Oxygen, Nitrogen and Sulphur content. Based on these properties, the species will have good heating values, efficient combustion, low handling and storage costs and low Nitrogen and Sulphur emissions. Their fixed carbon content also indicates that they will have good heating values. The low ash contents and volatile matter recorded makes them excellent raw materials for solid biofuel production. The calorific values (HHV and LHV) of the species makes them suitable for high energy-demanding applications. Their burning rates would be low. The Particulate Matter and Carbon Monoxide concentrations of Beema bamboo (90 ug/m^3^ and 2.83 ppm respectively) and *O*. *abyssinica* (77.33 ug/m^3^ and 3.20 ppm respectively) are lower than the allowable concentrations ((35000 ug/m^3^ and 9 ppm respectively) by the United States Environmental Protection Agency. Therefore, the species are good fuel materials safe for indoor use.

### Limitation of the study

The current work did not evaluate the NOx while burning biomass from the two species. This, however, did not affect the conclusions that were made in the work.

## Supporting information

S1 File (PDF)
