# Supplementary material for: Physico-thermal and emission properties of tissue cultured clone from Bambusa balcoaa (Beema bamboo) and Oxytenanthera abyssinica as sustainable solid biofuels
Source: PLoS One. 2022 Dec 22;17(12):e0279586. doi: 10.1371/journal.pone.0279586 (PMC9778976; doi:10.1371/journal.pone.0279586)
Supplement: S1 File — (PDF) [file pone.0279586.s001.pdf]

| Species | Density | Moisture c | Ash conten | Burning rate | HHV | LHV | Fixed carbo | Volatile ma | Particulate | Carbon mo | Organic car | Hydrogen |
|---|---|---|---|---|---|---|---|---|---|---|---|---|
| Beema bamboo | 0.43 | 7.5 | 3.45 | 3.53 | 23.12514 | 22.08702 | 28.11 | 60.94 | 86 | 2.9 | 52.69 | 10.1 |
| Beema bamboo | 0.48 | 7.8 | 3.65 | 3.45 | 23.05481 | 22.04296 | 28.57 | 59.98 | 95 | 3.1 | 52.75 | 9.79 |
| Beema bamboo | 0.39 | 7.6 | 2.4 | 3.57 | 23.47812 | 22.45128 | 28.39 | 61.61 | 89 | 2.5 | 52.8 | 9.97 |
| O. abyssinica | 0.52 | 7.37 | 2.8 | 2.56 | 23.35039 | 22.28991 | 27.12 | 62.71 | 78 | 3.1 | 53.13 | 10.35 |
| O. abyssinica | 0.49 | 7.04 | 2.75 | 2.5 | 23.36461 | 22.28861 | 27.46 | 62.75 | 72 | 3.7 | 52.97 | 10.55 |
| O. abyssinica | 0.56 | 7.5 | 3.6 | 2.4 | 23.07365 | 22.0023 | 26.2 | 62.7 | 82 | 2.8 | 52.8 | 10.45 |

| Nitrogen | Sulphur | Oxygen |
|---|---|---|
| 0.69 | 0.17 | 32.9 |
| 0.71 | 0.16 | 32.94 |
| 0.68 | 0.18 | 33.97 |
| 0.59 | 0.25 | 32.88 |
| 0.58 | 0.28 | 32.87 |
| 0.59 | 0.24 | 32.32 |
